# Trends and influencing factors of HIV health education receive rate among 0.57 million migrants in China from 2009 to 2017: a national population-based study

**DOI:** 10.1186/s12889-023-16140-6

**Published:** 2023-06-28

**Authors:** Yue Che, Jun Wang, Chao Song, Xueyao Wang, Yang Bai, Jue Liu

**Affiliations:** 1 Center for Health Policy Research and Evaluation, Renmin University of China, Beijing, 100872 China; 2 School of Public Administration and Policy, Renmin University of China, Beijing, 100872 China; 3 The First People’s Hospital of Anning City, Kunming, 650300 Yunnan Province, Yunnan Province China; 4 Department of Epidemiology and Biostatistics, School of Public Health, Peking University Health Science Centre, Beijing, 100080 China; 5 Institute for Global Health and Development, Peking University, Beijing, 100871 China; 6 Key Laboratory of Epidemiology of Major Diseases (Peking University), Ministry of Education, Beijing, 100191 China; 7 School of Public Health, Institute for Global Health and Development, Peking University, Peking University, Beijing, 100191 China

**Keywords:** BPHS, China, Migrant population, HIV, Health education, HIV health education rate

## Abstract

**Introduction:**

China has implemented Basic Public Health Service (BPHS) in 2009, aiming to improve the health status of the people, and the content of service includes implying health education for residents. As an important group of people, the migrants can easily become main reason for major infectious diseases such as HIV between different provinces, but the effect of receiving health education is still unknown for migrants. Therefore, the health education of China’s migrant population has received widespread attention.

**Methods:**

This study used the data of the China Migrants Dynamic Survey (CMDS) from 2009 to 2017, and evaluated the trend of HIV health education acceptance rate of different migrant groups across the country (n = 570,614). Logistic regression model was used to test the influencing factors of HIV health education rate. Results: The study found that the overall HIV health education rate of Chinese migrants decreased from 2009 to 2017, and different types of migrants showed different trends. The proportion of migrants aged 20–35 who receive education fluctuates, and ethnic minorities, western regions, and migrants with high education were more likely to receive HIV health education. Conclusion: These findings identify when implementing health education for migrants, we can carry out more education for specific groups to promote the health equity of the migrant population.

**Supplementary Information:**

The online version contains supplementary material available at 10.1186/s12889-023-16140-6.

## Introduction

In 2009, Chinese government began to implement Basic Public Health Services (BPHS). The goal of this service is to enable the Chinese people to achieve health equity as soon as possible [1]. In particular, we know that in the more developed regions of China, such as the eastern region, people’s living standards and per capita income are significantly higher than those in the central and western regions [2]. Accordingly, the education level and health awareness of the population in the eastern region are also stronger than those in the central and western regions [3]. BPHS is designed to enable people in the central and western regions of China’s economically underdeveloped regions to enjoy basic health services and improve the health level of the Chinese population [1]. The project contents and expenses from 2009 are in the appendix (Table 4).

By 2023, the expenditure of BPHS has been raised to 84 yuan/person/year. During the decades since 2009, Chinese government has adhered to this service year by year, and the financial input has continuously increased [4]. Till now, the government has paid a lot of human, financial, and material resources to achieve the equalization of health [5]. However, it is difficult to assess the impact of this service on health promotion, especially in the case of large migrant populations where health evaluation and health assessment are more difficult to achieve for migrants than for the resident population.

With the continuous development of China’s economy and society, the phenomenon of large-scale population migration and flow continues. In 2009, the total migrant population in China was 211 million people [6, 7]. By 2015, according to the National Bureau of Statistics of China, the total migrant population in China was 247 million people [8]. This expansion trend weakened after 2015, and the growth rate of China’s migrant population slowed down and entered a new stage. By the end of 2017, the total number of migrants was 244.5 million [8]. Therefore, health issues related to migrants have been widely concerned by academic circles.

The migrants are difficult to receive effective health management services due to its medical security system, household registration system, and health monitoring system [9]. In 2009, the Ministry of Health of China issued the “Opinions on Promoting the Gradual Equalization of Basic Public Health Services”, and providing basic public health services to all citizens, including the provision of health education publicity and consultation services to urban and rural residents, as well as health education Work [10]. Health education is currently available, easy to implement, well-applicable, and economical HIV prevention measures [11], in the form of community dissemination of publicity materials, radio/TV programs, knowledge lectures and training, short message/WeChat group chat, health consultation activities, peer education (based on the questionnaire), etc.

However, the health of Chinese migrants is not optimistic. In 2017, 54.5% of the migrant population’s health education status was below the standard, which is still far behind the target value of the migrant population’s health education coverage rate of > 95% by the relevant national departments in 2020 [7], which reminds the health education coverage of the migrant population in China The rate of health education and the level of health education are generally low, and health education services still need to be strengthened. In particular, some of the more vulnerable migrants, such as the elderly, as the key population of the country to implement basic public health service standards, are generally faced with problems such as poor health service utilization awareness and low utilization [12].

Especially in the face of highly harmful infectious diseases, immigration is considered to be an important factor in the spread of HIV [13]. For example, HIV is an infectious disease with rapid transmission and high mortality, which poses a serious threat to people’s health. In China and many other countries, immigrants may become HIV carriers among different populations. There are about 80% of HIV infections are in rural areas in China [14]. These migrants from rural areas have specific demographic and economic characteristics, such as lower education status and economic level lagging behind the migrant areas [6, 7]. In addition, due to the independence of cities and rural areas, household registration policies, medical security systems, etc. The restrictions on the migrants may lead to poor interaction between these migrants and the city, and suffer some social difficulties (isolation, separation from their families, marginalization, barriers to access to services, etc.) [15–18]. These conditions usually lead to frequent premarital or extramarital sex in this population [15, 19]. In China, sexual intercourse infection is the highest path of HIV infection, and more than 50% of HIV patients are infected through sexual intercourse [11, 20]. In the face of infectious diseases such as HIV, On the one hand, migration has become a priority group for HIV transmission due to their own conditions of the migrant group (age, education status, marital status, etc.), the household registration system, the medical security system, the health system, etc. [21]. On the one hand, due to the own conditions of the migrant group (age, education status, marital status, etc.), household registration system, medical security system, health system, and other restrictions. On the other hand, due to the increasing scope and quantity, migration has become an important factor contributing to the spread of HIV [22]. Therefore, it is particularly important to explore HIV health education policies and compaigns for migrant population, and to strengthen them in a targeted manner.

At present, it is not clear in the existing research whether the age of migrants, education status, the type of medical insurance, and the type of household registration is related to the acceptance rate of HIV health education among migrants. Therefore, we used the research data of the China Migrants Dynamic Survey (CMDS) to explore the uptake rates of HIV health education among migrant populations in different household registration areas, in different age groups and with different types of health insurance to better understand which populations are targeted by HIV. By 2019, the cumulative number of people infected with HIV has reached 38 million worldwide, and the goal of HIV prevention and control is difficult to achieve [23]. Because of this, greater benefits can be gained from more targeted HIV education for migrant populations.

## Materials and methods

### Data collection

We used data from China Migrants Dynamic Survey, which is a national panel database reflecting the basic situation of China’s migrant dynamic migration and health. This data is a public dataset obtained from the annual survey conducted by the Migrant Population Service Center, National Health Commission P.R. China (available website: https://www.ncmi.cn/phda/dataDetails.do?id=CSTR:A0006.11.A000T.201906.000225). Since 2009, the National Health Commission has conducted an annual large-scale nationwide sampling survey of mobile population, covering 32 provincial-level administrative regions across the country. We obtained survey results from four years: 2009, 2014, 2016, and 2017, and ultimately obtained the survey results of 0.57 million mobile population. The data content includes personal and family basic information, migration and mobility experience, social security status, medical and health services, marriage and childbirth, etc. The survey was randomly sampled in 31 provinces and Xinjiang Production and Construction Corps, and sampling points are selected from areas with a relatively concentrated migrants. Participants in the survey are those who have lived in the inflow area for more than one month and those who are not registered with residents and are aged 15 or above. The final sample data included 570,614 migrants.

## Measures

### Dependent variable

The main outcome variable was the question answered by the respondents who migrated to the current city: in the past year, have you received the following health education in your current village/residence (Yes or no). This question mainly reflects the health and public services received by migrant workers.

### Covariates

**Fig. 1:**
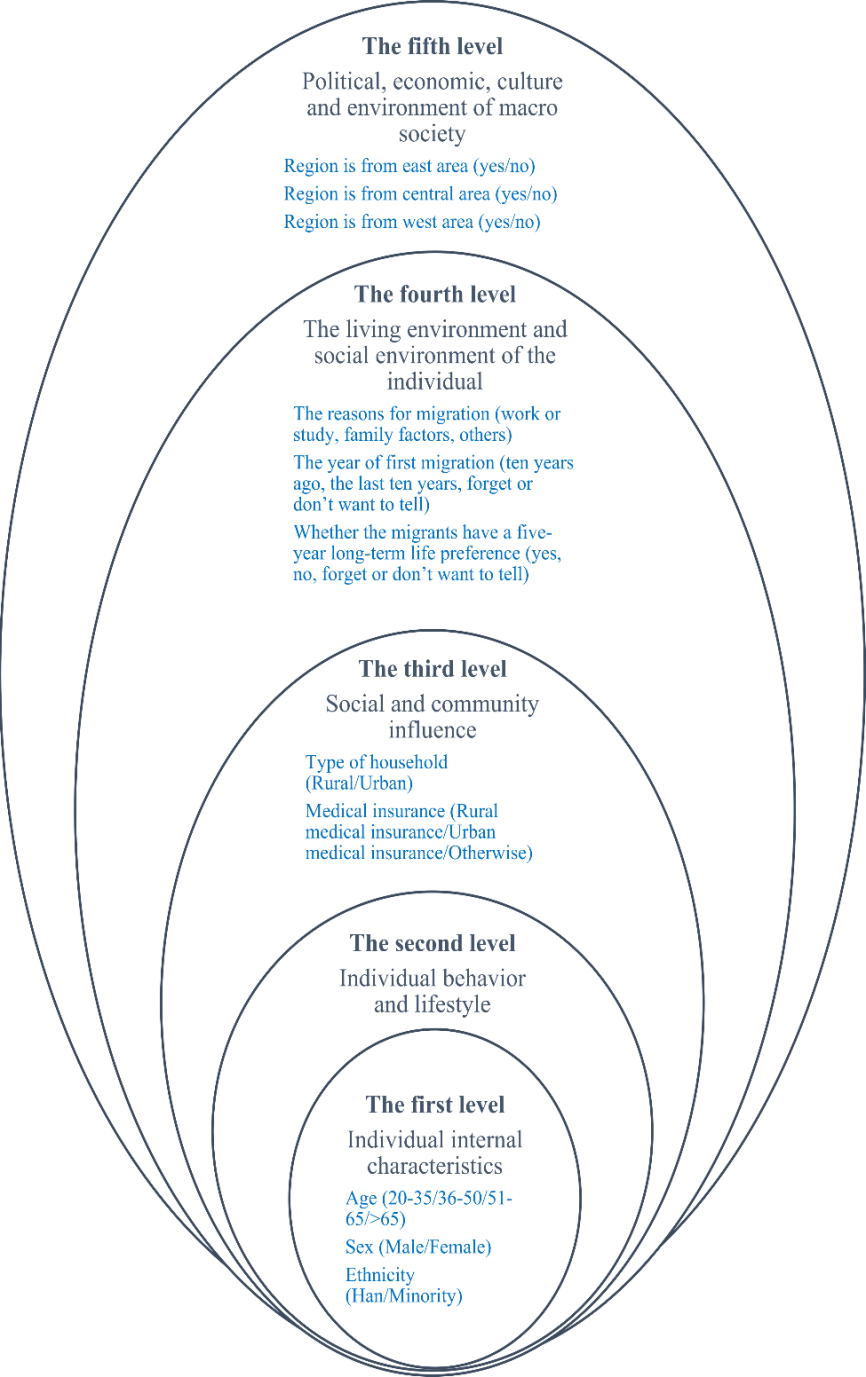
Model diagram of Social Determinants of Health and covatiates *≤Middle school: middle school or below *>College: higher than college

We use 11 variables as covariates. According to the theory of social determinants of health (SDH) (Fig. 1), due to people’s different social status and the environment determined by their resources, in addition to the factors that directly caused disease, other indirect factors are also closely related to people’s health level. Addressing the differences in SDH can help achieve health equity and provide everyone with the opportunity to achieve a higher level of health [24]. In the hierarchical model of health social factors established by Dahlgren and Whitehead in 1991, it is believed that five main factors will affect people’s health levels. The first layer represents individuals with different individual internal characteristics, the second layer represents individual behavior and lifestyle, the third layer represents the impact of society and community, the fourth layer represents living and working conditions, and the fifth layer represents the political, economic, cultural and environment of macro society.

In this study, we selected 11 covariates according to Dahlgren and Whitehead’s Dahlgren and Whitehead’s mode [25, 26]. The first level of individual characteristics includes age, gender, and nationality; The second level is individual behavior and lifestyle including marital status; Education status; The third level, social and community impact includes registered residence (urban or rural), Medical Insurance (Rural medical insurance, Urban medical insurance, otherwise); The fourth level, in this study, the living environment and social environment of individuals are mainly immigration-related variables, including the reasons for migration (work or study, family factors, others), the year of the first migration (ten years ago, the last ten years, forget or don’t want to tell), the five years since the current migration and the migrants have formatted a life preference (yes, no, forget or don’t want to tell). The fifth level is the political, economic, cultural, and environment of the macro society: the region (The eastern region includes 11 provincial administrative regions, namely Beijing, Tianjin, Hebei, Liaoning, Shanghai, Jiangsu, Zhejiang, Fujian, Shandong, Guangdong and Hainan; the central region includes eight provincial administrative regions, namely Heilongjiang, Jilin, Shanxi, Anhui, Jiangxi, Henan, Hubei and Hunan; the western region includes the Xinjiang Production and Construction Corps and 12 provincial administrative regions, namely Sichuan, Chongqing, Guizhou, Yunnan, Tibet, Shaanxi and Gansu, Qinghai, Ningxia, Xinjiang, Guangxi, Inner Mongolia).

### Statistical analysis

Descriptive statistical analysis data, including frequency and percentage, mean and standard deviation, were used to summarize and report demographic variables, individual economic variables, health and public services, and whether HIV health education was received. We used logistic regression models to investigate the trends of receiving HIV education among migrants from different regions. Independent factors related to HIV education were included in each of the four models. We used two models, one univariate and one multivariate regression. In the first model, we validated the impact of different years and demographic variables on HIV health education. In the second model, we validated region, education level, type of household, and medical insurance.

In all analyses, we considered that the *p* value less than 0.05 was statistically significant. Hosmer Lemeshaw were used to measure the goodness of fit of the logistic regression models. We used χ^2^ analyses to compare the covariates of each survey year to determine the statistically significant trend of each variable. We use SPSS version 25.0 (IBM, Armonk, NY) and Stata version 14.0 to conduct all the statistical analyses.

## Results

### Characteristics of the studied population

The sample characteristics included 570,614 migrants, including males (53.90%) and females (46.10%) (**Table **1). The average age was 42.11 years (SD = 10.34). Most of the participants were between 36 and 50 years old (47.55%), followed by migrants aged between 20 and 35 years old (30.88%). In general, 92.06% of them were Han ethnicity. There were 456,174 married migrants (79.90%). There were 134,325 (23.50%) migrants from the eastern region, 234,579 (41.10%) migrants from the central region, and 201,680 (35.30%) migrants from the western region. In the sample of migrants, more than half (60.60%) of them have an education in middle school or below, while the proportion of migrants with education in high school (23.40%), college (15.50%), and higher than college (0.60%) is relatively low. Most of the migrants were urban (81.70%) and less rural (18.30%). The number of migrants with urban medical insurance (38.90%) was higher than that with rural medical insurance (23.70%), and the number of migrants with other types of medical insurance accounted for about one-third (37.40%). In all the samples, the number of migrants who received HIV health education at the time of immigration (45.00%) was less than that of migrants who did not receive HIV health education (55.00%).

**Table 1:** Demographic characteristics and outcomes among migrants (N = 570,614)

	2009			2014			2016			2017			Overall	
	N	%		N	%		N	%		N	%		N	%
Gender***														
Male	13,772	44.60		117,647	58.50		88,059	52.10		87,799	51.70		307,277	53.90
Female	17,089	55.40		83,290	41.50		80,892	47.90		82,066	48.30		263,337	46.10
Age(years)***														
20–35	3,792	12.30		57,261	28.50		55,881	33.10		59,270	34.90		176,204	30.90
36–50	17,761	57.60		101,282	50.40		77,510	45.90		74,747	44.00		271,300	47.50
51–65	8,752	28.40		41,028	20.40		30,296	17.90		30,112	17.70		110,188	19.30
>65	556	1.80		1366	0.70		5,264	3.10		5,736	3.40		12,922	2.30
Ethnicity***														
Han	30,142	97.70		186,218	92.70		155,078	91.80		153,882	90.60		525,320	92.10
Minority	719	2.30		14,719	7.30		13,873	8.20		15,983	9.40		45,294	7.90
Marital status***														
Married	25,413	82.30		153,789	76.50		137,418	81.30		139,553	82.20		456,174	79.90
Otherwise	5448	17.70		47,147	23.50		31,533	18.70		30,312	17.80		114,440	20.10
Education status***														
≤Middle school	4,726	15.30		133,812	66.60		104,275	61.70		103,138	60.70		345,951	60.60
High school	17,108	55.40		41,289	20.50		37,670	22.30		37,187	21.90		133,254	23.40
College	8,127	26.30		25,183	12.50		26,200	15.50		28,650	16.90		88,160	15.50
>College	900	2.90		653	0.30		806	0.50		890	0.50		3,249	0.60
Type of household***														
Rural	25,689	83.20		169,061	84.10		138,845	82.20		132,555	78.00		466,150	81.70
Urban	5,172	16.80		31,876	15.90		30,106	17.80		37,310	22.00		104,464	18.30
Medical insurance***														
Rural medical insurance	0	0.00%		3,458	1.70%		110,769	65.60%		107,457	63.30%		221,684	38.90%
Urban medical insurance	2,559	8.30%		46,492	23.10%		37,910	22.40%		48,302	28.40%		135,263	23.70%
Otherwise	28,302	91.70%		150,987	75.10%		20,272	12.00%		14,106	8.30%		213,667	37.40%
Reason for migration***														
Work or study	18,200	58.97%		3,173	1.57%		25,305	14.98%		26,597	15.66%		73,275	12.84%
Family factors	5,237	16.97%		196,307	97.70%		141,193	83.57%		142,065	83.63%		484,802	84.96%
Others	7,424	24.06%		1,457	0.73%		2,453	1.45%		1,203	0.71%		12,537	2.20%
Year of first migration***														
Ten years ago	2,762	8.94%		62,284	31.00%		17,510	10.36%%		75,114	44.22%		157,670	27.63%
The last ten years	3,218	10.40%		138,653	69.00%		15,725	9.31%		94,326	55.53%		251,922	44.15%
Forget or don’t want to tell	24,881	80.66%		0	0.00%		135,716	80.33%		425	0.25%		161,022	28.22%
≥ 5 years migration and have a life preference now***														
Yes	4,405	14.27%		23,379	12%		26,954	15.95%		33,567	19.76%		88,305	15.48%
No	26,435	85.66%		177,558	88%		141,997	84.05%		13,6298	80.24%		482,288	84.52%
Forget or don’t want to tell	21	0.07%		0	0.00%		0	0.00%		0	0.00%		0	0.00%
Region***														
East area	8,064	26.10%		45,596	22.70%		40,651	24.10%		40,014	23.60%		134,325	23.50%
Central area	13,439	43.50%		85,096	42.30%		67,642	40.00%		68,432	40.30%		234,579	41.10%
West area	9,358	30.30%		7,0245	35.00%		60,658	35.90%		61,419	36.20%		201,680	35.30%
Received HIV education***														
Yes	19,955	64.70%		90,038	44.80%		85,517	50.60%		61,233	36.00%		256,743	45.00%
No	10,906	35.30%		110,892	55.20%		83,434	49.40%		108,632	64.00%		313,871	55.00%

*≤Middle school: middle school or below

*>College: higher than college

### HIV education receiver characteristics

The average proportion of the migrant population receiving HIV education during the four years was 45.0% (**Table **1). There are obvious differences in the demographic and sociological characteristics of the migrant population in different years (all P < 0.001) (**Table **2).

**Table 2:** The rate of received HIV education of migrates in China by region, gender, age, marriage, and education status of 2009, 2014, 2016, 2017

	N		Received HIV education (%)									X^2^		P
			Overall		2009		2014		2016		2017			
**Gender**												2288.69		< 0.001
Male	307,277		136,732(44.50)		8,239(59.82)		52,666(44.77)		44,247(50.25)		31,580(35.97)			
Female	263,337		120,011(45.57)		11,716(68.56)		37,372(44.87)		41,270(51.02)		29,653(36.13)			
**Age(years)**												6470.32		< 0.001
20–35	176,204		77,869(44.19)		2,130(56.17)		25,693(44.87)		28,774(51.49)		21,272(35.89)			
36–50	271,300		127,286(46.92)		11,861(66.78)		46,603(46.01)		40,134(51.78)		28,688(38.38)			
51–65	110,188		475,18(43.12)		5,679(64.89)		17,278(42.11)		14,689(48.48)		9,872(32.78)			
>65	12,922		4,070(31.50)		285(51.26)		464(33.97)		1,920(36.47)		1,401(24.42)			
**Ethnicity**												1653.96		< 0.001
Han	525,320		233,317(44.41)		19,523(64.77)		82,574(44.34)		76,795(49.52)		54,425(35.37)			
Minority	45,294		23,426(51.72)		432(60.08)		7,464(50.71)		8,722(62.87)		6,808(42.60)			
**Marital status**												1392.76		< 0.001
Married	456,174		205,739(45.10)		16,918(66.57)		68,779(44.72)		69,310(50.44)		50,732(36.35)			
Otherwise	114,440		51,004(44.57)		3,037(55.75)		21,259(45.09)		16,207(51.40)		10,501(34.64)			
**Region**												529.11		< 0.001
East area	134,325		55,223(41.11)		4,813(59.69)		19,527(42.83)		18,199(44.77)		12,684(31.70)			
Central area	234,609		97,944(41.75)		8,046(59.87)		35,792(42.06)		31,467(46.52)		22,639(33.08)			
West area	201,680		103,576(40.30)		7,096(75.83)		34,719(49.43)		35,851(59.10)		25,910(42.19)			
**Education status**												18226.82		< 0.001
≤Middle school	345,951		147,173(42.54)		2,976(62.97)		57,094(42.67)		52,159(50.02)		34,944(33.88)			
High school	133,254		64,521(48.42)		10,796(63.10)		19,327(46.81)		19,695(52.28)		14,703(39.54)			
College	88,160		43,407(49.24)		5,573(68.57)		13,250(52.61)		13,269(50.65)		11,315(39.49)			
>College	3,249		1,642(50.54)		610(67.78)		367(56.20)		394(48.88)		271(30.45)			
**Type of household**												797.16		< 0.001
Rural	466,150		208,433(44.71)		16,347(63.63)		74,455(44.04)		70,287(50.62)		47,344(35.72)			
Urban	104,464		48,310(46.25)		3,608(69.76)		15,583(48.89)		15,230(50.59)		13,889(37.23)			
**Medical insurance**												144679.92		< 0.001
Rural medical insurance	221,684		95,711(43.17)		0(0.00)		1,490(43.09)		56,244(50.78)		37,977(35.34)			
Urban medical insurance	135,263		65,141(48.16)		1,952(76.28)		23,976(51.57)		20,057(52.91)		19,156(39.66)			
Otherwise	213,667		95,891(44.88)		18,003(63.61)		64,572(42.77)		9,216(45.46)		4,100(29.07)			
**Total**	570,614		256,743(44.99)		19,955(64.66)		90,038(44.81)		85,517(50.62)		61,233(36.05)			

*≤Middle school: middle school or below

*>College: higher than college

**Fig. 2:**
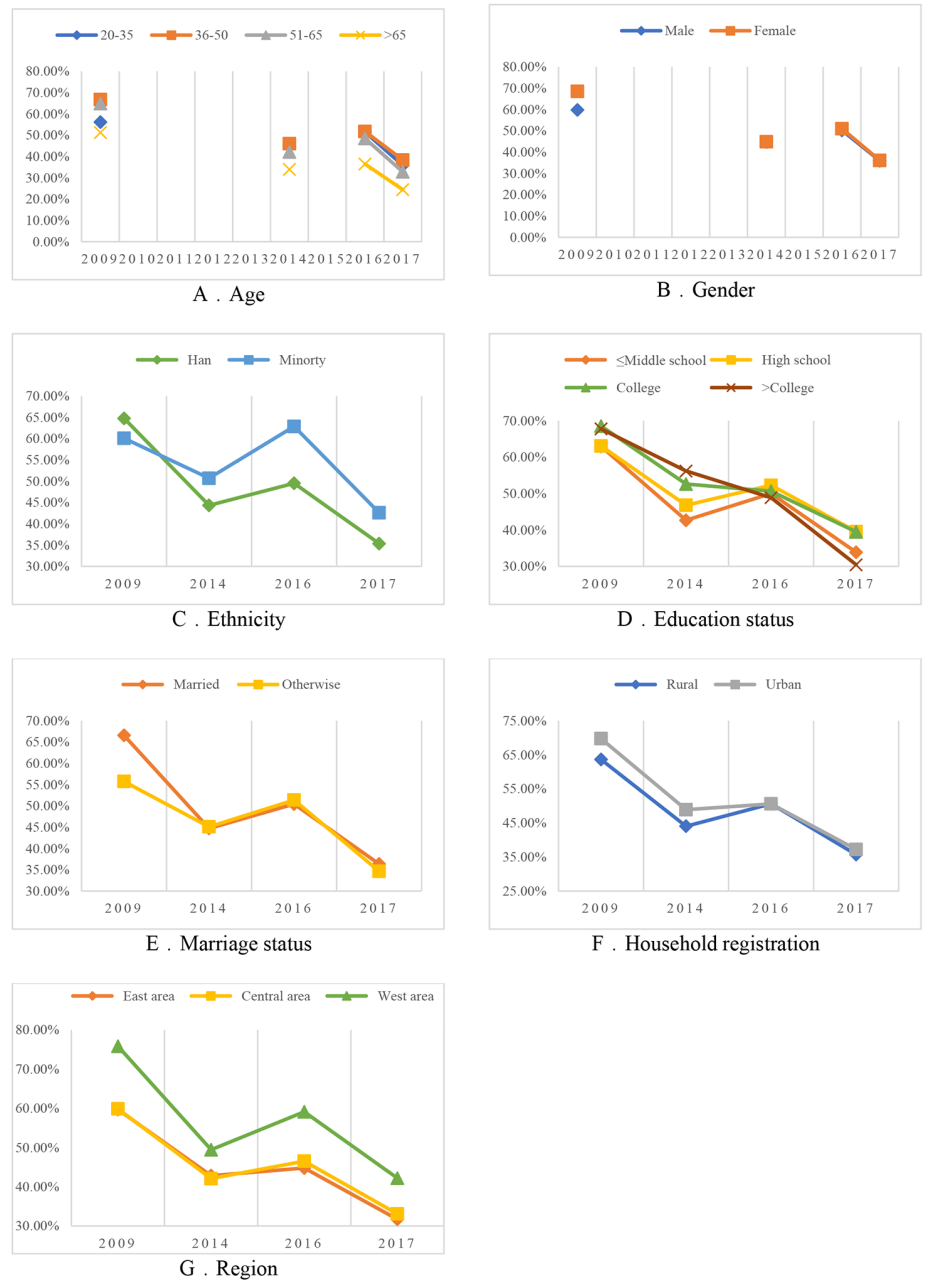
Trends in the proportion of HIV health education among different types of migrants of 2009, 2014, 2016, 2017 *≤Middle school: middle school or below *>College: higher than college

According to the model of SDH at the first level, the proportion of migrants who have received HIV health education aged 36 to 50 is the highest among different age groups (Fig. 2. A). but since 2014, the proportion of people aged 20–35 increased to 44.87%, just a little bit lower than migrants aged 36–50. The proportion of people aged 51–65 receiving HIV education is slightly lower than that of youth and middle-aged groups. In different genders, the proportion of female migrants receiving HIV education (68.56%) was significantly higher than that of men in 2009 (59.82%) (Fig. 2B). And for migrants of different ethnicities, the proportion of Han migrants receiving HIV health education in 2009 (64.77%) was higher than that of ethnic minority migrants (60.08%), but in the following three years of data, the proportion of ethnic minority migrants far exceeded that of Han migrants (Fig. 2 C).

Besides, the group with the highest level of education, who is above college degree, gradually decreased from the highest proportion of people receiving HIV education (68.57%) to 30.45% (Fig. 2D). The proportion of migrants with college degree has also gradually decreased from 67.78% to 2009 to 39.49%. The change in the proportion of ‘high school’ and ‘less than middle school’ receiving HIV education is consistent with the changing trend of the overall proportion. In addition, the proportion of married migrants receiving HIV education was (66.57%), which was significantly higher than that of unmarried migrants (55.75%) (Fig. 2E).

From the third level to the fifth level, it’s worth mentioning the aspects of household and region. In Fig. 2. F, in 2009 and 2014, the proportion of migrants from urban and rural areas receiving HIV health education was relatively high (69.76% and 48.89%), but in 2016 and 2017, the proportion of urban migrants receiving HIV education picked up (50.59% and 37.23%), which was comparable to that of urban and rural migrants receiving HIV health education (50.26% and 35.72%). During the four years, the proportions of migrants from the western region who received HIV education in these four years were 75.83%, 49.43%, 59.10%, and 42.19% (Fig. 2G), significantly more than those from the central and eastern regions. In 2016 and 2017, the proportion of migrants in the central region (46.52% and 33.08%) receiving HIV education was slightly higher than that in the eastern region (44.77% and 31.70%).

### Logistic regression model

Compared with men, women are slightly more likely to receive HIV health education, but there is little difference between the two (OR = 1.031, 95% CI: 1.020–1.042, **Table **3). Compared with young people, middle-aged people are more likely to receive HIV health education (OR = 1.066, 95% CI: 1.051–1.081), while elderly people over 65 are less likely to receive HIV health education (OR = 0.562, 95% CI: 0.540–0.585). Ethnic minorities may receive more HIV health education than Han people (OR = 1.211, 95% CI: 1.187–1.236). Unmarried and other people may receive less health education than married (OR = 0.912, 95% CI: 0.898–0.926). Migrant in the central region are slightly more likely to receive HIV health education than in the east (OR = 1.076, 95% CI: 1.061–1.091), and migrants in the western region are significantly higher than those in the eastern region (OR = 1.590, 95% CI: 1.567–1.613). Univariate regression model shows that compared with migrants below middle school, migrants with higher education are more likely to receive HIV health education. Compared with the rural household registration floating population, the urban household registration population is slightly less likely to receive HIV health education (OR = 1.064, 95% CI: 1.050–1.078). Multivariate regression model shows that compared with rural medical insurance, urban medical insurance (OR = 1.148, 95% CI: 1.129–1.168) and other types of medical insurance (the possibility of receiving HIV is higher (OR = 0.819, 95% CI: 0.804–0.834).

**Table 3:** Univariate analysis and multivariate regression related to received HIV education among migrants, CMDS of 2009, 2014, 2016, 2017

	univariate regression model										multivariate regression model						
	OR (95% CI)		p									OR(95%CI)		p			
Year (compared to 2017)																	
2009	3.246 (3.165–3.329)		< 0.001									3.838 (3.724–3.955)			< 0.001		
2014	1.440 (1.421–1.460)		< 0.001									1.682 (1.652–1.713)			< 0.001		
2016	1.818 (1.794–1.844)		< 0.001									1.874 (1.848-1.900)			< 0.001		
Gender (compared to male)	1.044(1.034–1.055)		< 0.001									1.031 (1.020–1.042)			< 0.001		
Age (compared to 20–35)																	
36–50	1.116 (1.103–1.130)		< 0.001									1.066 (1.051–1.081)			< 0.001		
51–65	0.958 (0.943–0.972)		< 0.001									0.923 (0.907–0.940)			< 0.001		
>65	0.581 (0.559–0.603)		< 0.001									0.562 (0.540–0.585)			< 0.001		
Ethnicity (compared to Han)	1.341 (1.315–1.367)		< 0.001									1.211 (1.187–1.236)			< 0.001		
Marital status (compared to married)	0.979 (0.966–0.992)		0.001									0.912 (0.898–0.926)			< 0.001		
Region (compared to East area)																	
Central area	1.027 (1.013–1.041)		< 0.001									1.076 (1.061–1.091)			< 0.001		
West area	1.512 (1.491–1.534)		< 0.001									1.590 (1.567–1.613)			< 0.001		
Education status (compared to Middle school or below)																	
High school	1.268 (1.252–1.284)		< 0.001									1.067 (0.992–1.148)			< 0.001		
College	1.310 (1.291–1.330)		< 0.001									0.912 (0.898–0.926)			< 0.001		
Higher than college	1.380 (1.288–1.479)		< 0.001									1.076 (1.061–1.091)			0.081		
Type of household (compared to rural)	1.064 (1.050–1.078)		< 0.001									0.987 (0.971–1.002)			0.094		
Medical insurance (compared to rural)																	
Urban medical insurance	1.223 (1.206–1.239)		< 0.001									1.148 (1.129–1.168)			< 0.001		
Otherwise	1.072 (1.059–1.085)		< 0.001									0.819 (0.804–0.834)			< 0.001		

## Discussion

As far as we know, there was little study to examine the proportion of the migrant population in China receiving HIV education based on data from the national population. We discussed the differences in the acceptance of HIV health education among migrants of different gender, ages, ethnicity, marital status, region place, education status, types of household registration, types of medical insurance, etc. Through analysis, we found that: it may be due to the inconsistency of population characteristics, and the implementation of HIV health education is affected by the characteristics of migrants, resulting in different age groups, different ethnicities, different marital statuses, different regions, and different education status, which causes the migrants to have different possibilities to receive HIV health education.

We found that there were significant differences in the proportion of registered residence migrants receiving HIV education in different regions of China. Compared with the central Eastern and western regions, the proportion of migrants receiving HIV health education in the western region is higher than that in other regions. In addition, over time, the proportion of HIV-educated migrants in the central region is slightly higher than that in the eastern region in recent years, which is consistent with the research results of Shtarkshall [13].

In addition, we also found that the increase in the proportion of ethnic minority migrant groups receiving HIV health education after 2009 may be related to regional factors. The top seven provinces with the largest proportion of ethnic minorities in China are: Tibet, Xinjiang, Qinghai, Guangxi, Guizhou, Yunnan, and Ningxia, and these provinces are all western regions. As HIV health education is widely implemented in the western region [13], the proportion of ethnic minority migrants receiving HIV health education has also increased.

According to the results, the proportion of HIV education varies with age. Over time, the migrant population receiving HIV health education has gradually become younger, and the proportion of the low-age population receiving HIV education has been increasing. This may be related to the following reasons: Firstly, in China, the most important way of transmission of HIV is through sexual transmission [20, 27, 28]. The groups who transmit HIV through sexual channels are younger than other channels. With the advancement of adolescent sexual maturity, the age of adolescents’ first sexual intercourse is decreasing. According to data from the “China Family Development Report 2015“[29] (30), the age of Chinese adolescents at first sex is 15.9 years old. Therefore, the proportion of young people receiving HIV health education among the floating population has increased. Secondly, the educated population is younger. China began to implement nine-year compulsory education in 1986, allowing more people to receive secondary and higher education. In the 20th century, the proportion of young people with higher education and higher education has increased. These people have higher health awareness and health education status (31), and they have a deeper understanding of HIV prevention and treatment knowledge. Therefore, the proportion of young migrants receiving HIV health education is increasing.

We also found that changes in the proportion of HIV education were related to educational status. In addition, the proportion of HIV health education received by migrants is also related to the level of education. In the 1990s, Chinese migrants were mainly people with lower educational backgrounds, most of whom were at the elementary school level or even illiterate [32]. With the popularization of nine-year compulsory education and the continuous development of education in China, the education status of the main group of migrants has improved, mainly with junior high school education [8, 32].

There were several limitations. In the data obtained in this study, there are few social and economic measurement indicators for individuals, and only the individual’s marital status, education status, household registration category, and medical insurance category are included. More indicators, such as monthly income, medical records, immigration willingness, immigration duration, and other information, due to the different information obtained in the survey of different years, no relevant information was obtained in the survey of some years, and the availability of data was missing Inferior, so more individual socio-economic indicators are not discussed in this study. Although the article selects four-year cross-sectional data, which describes the HIV education situation and health level of migrants to a certain extent, the existing data is not consecutive year panel data and cannot fully reflect the time continuity. The data in this article can only explain the trend of data changes, and no further causal research can be conducted.

## Conclusion

We studied HIV health education and its influencing factors, including gender, age, ethnicity, marital status, region of place, education status, registered residence type, and medical insurance type. The study found that the proportion of migrants receiving HIV health education will be different in different age groups, regions, ethnicity, and education status. Therefore, when implementing HIV health education, the government and health institutions can give more consideration to strengthening publicity and education for specific groups, enhancing the health awareness of migrants, and generating more benefits from health education.

## Electronic supplementary material

Below is the link to the electronic supplementary material.

**Appendix: Table. 4** The items and expenses of BPHS from 2009-2022

## Data Availability

The dataset supporting the conclusions of this article was acquired at https://www.ncmi.cn/phda/dataDetails.do?id=CSTR:A0006.11.A000T.201906.000225.

## Abbreviations

HIV: Human immunodeficiency virus
CDMS: China Migrants Dynamic Survey
STDs: Sexually transmitted disease
