# Supplementary material for: Trends and influencing factors of HIV health education receive rate among 0.57 million migrants in China from 2009 to 2017: a national population-based study
Source: BMC Public Health. 2023 Jun 28;23:1256. doi: 10.1186/s12889-023-16140-6 (PMC10308794; doi:10.1186/s12889-023-16140-6)
Supplement: Supplementary file 1 — Appendix: Table. 4 The items and expenses of BPHS from 2009-2022 [file 12889_2023_16140_MOESM1_ESM.docx]

**Appendix**

**Table.4 The items and expenses of BPHS from 2009-2022**

| **year** | **BPHS items** | **Expenditures**  **(Yuan per person)** |
| --- | --- | --- |
| 2009 | Health file management, health education, 0-36 month children's health management, maternal health management, elderly health management, vaccination, infectious disease report and treatment, hypertension and diabetes patients' health management, severe mental disorder patients' management | 15 |
| 2011 | **Health and family planning supervision and management** | 25 |
| 2013 | Health management of traditional Chinese medicine for the elderly and children | 30 |
| 2015 | The health management of tuberculosis patients | 40 |
| 2017 | Free contraceptives and health literacy promotion action | 50 |
| 2019 | Major public health service projects are included in basic public health service projects (including endemic disease prevention, occupational disease prevention, major disease and health hazard monitoring, human avian influenza, SARS prevention and control projects, pestis prevention and control, health literacy promotion projects, etc.) | 69 |
| 2020 | Daily COVID-19 prevention and control | 74 |
| 2022 | Basic public health services and epidemic prevention and control in primary medical and health institutions | 84 |
